# Pressure overload leads to an increased accumulation and activity of mast cells in the right ventricle

**DOI:** 10.14814/phy2.13146

**Published:** 2017-03-22

**Authors:** Himal Luitel, Akylbek Sydykov, Yves Schymura, Argen Mamazhakypov, Wiebke Janssen, Kabita Pradhan, Astrid Wietelmann, Djuro Kosanovic, Bhola Kumar Dahal, Norbert Weissmann, Werner Seeger, Friedrich Grimminger, Hossein Ardeschir Ghofrani, Ralph Theo Schermuly

**Affiliations:** ^1^ Excellence Cluster Cardio‐Pulmonary SystemUniversities of Giessen and Marburg Lung CenterMember of the German Lung CenterJustus‐Liebig‐University GiessenGiessenGermany; ^2^ Department of Lung Development and RemodellingMax‐Planck Institute for Heart and Lung ResearchBad NauheimGermany; ^3^ Max‐Planck Institute for Heart and Lung ResearchMRI Service GroupBad NauheimGermany

**Keywords:** Mast cell, pulmonary artery banding, right ventricular hypertrophy

## Abstract

Right ventricular (RV) remodeling represents a complex set of functional and structural adaptations in response to chronic pressure or volume overload due to various inborn defects or acquired diseases and is an important determinant of patient outcome. However, the underlying molecular mechanisms remain elusive. We investigated the time course of structural and functional changes in the RV in the murine model of pressure overload‐induced RV hypertrophy in C57Bl/6J mice. Using magnetic resonance imaging, we assessed the changes of RV structure and function at different time points for a period of 21 days. Pressure overload led to significant dilatation, cellular and chamber hypertrophy, myocardial fibrosis, and functional impairment of the RV. Progressive remodeling of the RV after pulmonary artery banding (PAB) in mice was associated with upregulation of myocardial gene markers of hypertrophy and fibrosis. Furthermore, remodeling of the RV was associated with accumulation and activation of mast cells in the RV tissue of PAB mice. Our data suggest possible involvement of mast cells in the RV remodeling process in response to pressure overload. Mast cells may thus represent an interesting target for the development of new therapeutic approaches directed specifically at the RV.

## Introduction

Right ventricular (RV) remodeling represents a complex set of functional and structural adaptations induced by chronic pressure or volume overload due to various inborn defects or acquired diseases. Although the response of the RV to chronic pressure overload is an important determinant of patient outcome, the underlying molecular mechanisms remain elusive (Guarracino et al. 2005). A better understanding of the mechanisms of the RV remodeling may help identify candidate targets for novel therapeutic strategies directed specifically at the RV and thus improve survival in these patients. The availability of a murine model of pulmonary artery banding (PAB) provides us with a valuable tool to explore the mechanisms involved in the RV remodeling following pressure overload (Rockman et al. 1994; Tarnavski et al. 2004; Tarnavski 2009). Recently, a serial echocardiographic assessment of RV function changes in FVB mice subjected to PAB has been reported (Urashima et al. 2008). However, a more detailed investigation of longitudinal changes in concomitant maladaptive processes, including cardiomyocyte hypertrophy, myocardial fibrosis, and capillary density alterations, was not performed. In addition, numerous studies have demonstrated that significant differences in cardiac structure and function exist between mouse strains (Shah et al. 2010; Moreth et al. 2014). Even when cardiovascular parameters may not be different between strains under physiological conditions, genetic background may affect the cardiovascular response to various insults, such as hypoxia, aortic constriction, ischemia‐reperfusion, or myocardial infarction (Campen et al. 2004; Gao et al. 2005; Barrick et al. 2007; Izikki et al. 2007; Tada et al. 2008; van den Borne et al. 2009; Barnabei et al. 2010). Moreover, strain‐specific differences may affect results of pharmacological treatments (Lucki et al. 2001). Therefore, a direct comparison of findings of studies in which different inbred strains have been used may be challenging or impossible.

As recently shown in the rat SU/Hypoxia model of pulmonary arterial hypertension, differences in background strain can also affect the ability of the right heart to cope with increased pulmonary artery pressure (Jiang et al. 2016). However, the interstrain differences in the response of the RV to pressure overload have not been investigated comprehensively yet. Interestingly, it has recently been shown that C57BL/6J, the most widely used strain in the biomedical research (Simon et al. 2013), and FVB mice have different cardiovascular phenotypes at baseline and in response to acute hypoxic challenge (Barnabei et al. 2010). As a detailed characterization of the time course of pressure overload‐induced changes in RV morphology and function in other commonly used mouse strains is still lacking, we sought to investigate serial changes in RV morphology and function in C57BL/6J mice subjected to sustained pressure overload.

Although mast cell functions have been related to immune responses, a rapidly growing body of evidence has implicated these cells in a large variety of pathophysiologic processes (Puxeddu et al. 2003). Mast cells are also present in the normal heart tissues in humans and animals (Marone et al. 2000) and an increasing number of studies suggest possible roles of cardiac mast cells in the pathogenesis of various cardiovascular diseases (Levick et al. 2011). In particular, enhanced accumulation of mast cells in hypertensive and failing left ventricles has been documented (Panizo et al. 1995; Shiota et al. 2003; Batlle et al. 2006). Recent studies have identified important roles for mast cells in left ventricular hypertrophy and failure (Hara et al. 2002; Kitaura‐Inenaga et al. 2003; Stewart et al. 2003). However, the role of mast cells in the development of RV hypertrophy is poorly understood. The goal of this study was therefore twofold. First, to better understand longitudinal changes in RV morphology and function in C57BL/6J mice subjected to sustained pressure overload. Second, to investigate the involvement of mast cells in the RV remodeling process.

## Material and Methods

8–10‐week‐old male C57BL/6J mice (Charles River Laboratories, Sulzfeld, Germany) were maintained under appropriate barrier conditions in a 12‐h/12‐h light‐dark cycle and received standard laboratory food ad libitum and free access to water. All procedures involving animals were approved by the governmental Animal Ethics Committee (Regierungspräsidium Darmstadt).

### Model of PAB

Pressure overload was induced by PAB (*n* = 10 mice for each time point) as described (Kreymborg et al. 2010). Briefly, buprenorphine hydrochlorid (0.05 mg/kg bw, Vetergesic, Braun) was administered s.c. as an analgesic prior to operation. Surgery was performed under general anesthesia using isoflurane (1.5–2.5% v/v). The animals were placed on a heating pad to maintain body temperature and were ventilated with a rodent ventilator (MiniVent Type 845, Hugo Sachs Elektronik KG, March, Germany). A lateral thoracotomy was performed to gain access to the pulmonary artery. The pulmonary artery was constricted to 350 *μ*m using titanium clips (Hemoclip^®^, Weck, Germany) and a modified, adjustable clip applier (Hemoclip^®^). The chest and the skin incisions were closed by standard surgery techniques. Sham‐operated mice (*n* = 10 mice for each time point) served as controls and underwent an identical procedure except for the placement of the clip around the pulmonary artery. Carprofen (8 mg/kg per day, Rimadyl^®^, Pfizer) was administered via drinking water for 3 days post operation. Thereafter, animals were observed for various time periods after surgical procedures.

### Magnetic resonance imaging

Cardiac magnetic resonance imaging (MRI) was performed at baseline (before surgery) and on postoperative days 3, 7, 14, and 21 (*n* = 10 mice per group for each time point). Mice were anesthetized by inhalation of isoflurane (2% v/v) in oxygen/medical air (0.5/0.5 L/min) mixture. Cardiac MR measurements were performed on a 7.0T Bruker PharmaScan, equipped with a 300 mT/m gradient system, using a custom‐built circularly polarized birdcage resonator and the IntraGate^™^ self‐gating tool (Bruker, Ettlingen, Germany). The measurement is based on the gradient echo method (repetition time = 6.2 msec; echo time = 1.6 msec; field of view = 2.20 × 2.20 cm; slice thickness = 1.0 mm; matrix = 128 × 128; repetitions = 100; resolution 0.0172 cm/pixel). The imaging plane was localized using scout images showing the sagittal and coronal views of the heart, followed by acquisition in axial view, orthogonally to the septum of both scout scans. Multiple (9–10) contiguous axial slices were acquired for complete coverage of the ventricles. MRI data were analyzed using MASS4Mice digital imaging software (Medis, Leiden, Netherlands). The RV and left ventricular end‐diastolic volume (EDV) and end‐systolic volume (ESV) were determined and based on these values stroke volume (SV = EDV‐ESV), ejection fraction (EF = (SV/EDV)*100%), and cardiac output (CO = SV × heart rate) were calculated automatically. The myocardial volume was determined as the area inside the epicardial border minus the endocardial area. The myocardial mass was then obtained by multiplying the myocardial volume and the density of myocardial tissue (1.05 g/mL). Left ventricular eccentricity index (LVEI) values were calculated for both end‐diastole and end‐systole in the axial plane at midpapillary level. It is calculated as the ratio of the length of the major axis of the left ventricle (L1), which runs parallel to the septum, to the length of the minor axis of left ventricle (L2), which runs perpendicular to the septum: LVEI = L1/L2.

### Hemodynamic measurements

In vivo hemodynamic measurements were performed after completion of the protocols to induce RV hypertrophy (*n* = 10 mice per group for each time point). Mice were anesthetized using isoflurane (1.5% v/v) and placed on controlled heating and the core temperature, measured via rectal probe, was maintained at 37°C. The right jugular vein was used for catheterization of the RV to measure RV systolic pressure (RVSP), RV end‐diastolic pressure (RVEDP), and time constant of isovolumic pressure decay Tau. Systemic arterial pressure (SAP) was measured by catheterizing the right carotid artery. Hemodynamic measurements were performed using a Millar microtip catheter (SPR‐671, FMI, Foehr Medial Instruments GmbH, Seeheim/Ober‐Beerbach, Germany) and a PowerLab 8/30 System with the Chart 7.0 Software (AD Instruments GmbH, Spechbach, Germany).

### Tissue processing and histology

Hearts were harvested immediately after animals were killed (*n* = 5 mice per group for each time point). The ventricles were dissected free of the great vessels and atria. The RV was isolated from the left ventricle (LV) + septum (LV + S) by dissection along the septal insertion. The RV and (LV + S) were patted dry and weighed. The RV hypertrophy was assessed using the ratio of the RV mass to tibia length and to body weight.

Freshly dissected RV tissue was fixed in 4% paraformaldehyde overnight, then dehydrated and embedded in paraffin and sectioned at a thickness of 3 *μ*m. To detect collagen fibers, RV sections were stained with 0.1% sirius red (sirius red F3B, Niepoetter, Bürstadt, Germany) in picric acid (Fluka, Neu‐Ulm, Germany). For cardiomyocyte size determination transverse sections of the RV were stained with FITC conjugated wheat germ agglutinin (WGA‐FITC, Sigma‐Aldrich, Steinheim, Germany) for membrane staining. For quantification of the capillaries, sections were stained with IB4‐TRITC (Sigma Aldrich). Nuclei were stained with 4′,6‐diamidino‐2‐phenylindole (DAPI, Invitrogen, Karlsruhe, Germany) and mounted in DAKO fluorescence mounting medium (DAKO, Hamburg Germany). Sections without WGA‐FITC, IB4‐TRITC, and DAPI staining were used as a negative control. Photomicrographs were quantified to determine the mean cross‐sectional area of cardiomyocytes and interstitial collagen fraction using Leica Qwin V3 computer‐assisted image analysis software (Leica Microsystem, Wetzlar, Germany). Photomicrographs were quantified to count the number of cardiomyocytes and capillaries using STEPanizer image analysis tool (University of Bern, Department of Anatomy, Bern, Switzerland). Myocardial capillary density was expressed as a number of capillaries per cardiomyocyte. Average data reflect results from at least four or five different hearts in each group (more than 100 cells for each heart).

### Mast cell density and activity

To identify mast cells, toluidine blue staining was performed using standard protocol (Dahal et al. 2011). Briefly, paraffin‐embedded RV tissue sections were dewaxed, rehydrated and incubated with 0.05% w/v toluidine blue for 2–3 min. Mast cell density was quantified by counting the number of toluidine blue‐positive cells from each entire RV longitudinal sections (Leica QWin, 200X) in two random sections. The mast cell density was expressed as a number of mast cells per mm^2^. Degranulated mast cells are identified as cells in which granules are substantially reduced by 70–90% (Kanellakis et al. 2012). The results were expressed as percentage of degranulated mast cell to total amount of mast cells.

### Quantitative real‐time PCR

RV tissue samples were harvested immediately after mice were killed, snap‐frozen in liquid nitrogen and stored at −80°C until further analysis (*n* = 5 mice per group for each time point). Total mRNA was extracted from snap‐frozen mouse RV tissue samples using RNeasy Mini Kit (Qiagen, Hilden, Germany) according to the manufacturer's protocol. The primers used for real‐time quantitative PCR are presented in the Table 1. All results were normalized to the relative expression of the constitutively expressed gene porphobilinogen deaminase (PBGD). The relative transcript abundance of the target gene is expressed in ΔC_*t*_ values (ΔC_*t*_ = C_*t*_ reference–C_*t*_ target).

**Table 1:** Sequence of the primers used in quantitative real‐time PCR reactions

Gene	Primer sequence
ANP	Forward Primer: TCTGCCCTCTTGAAAAGCAA Reverse Primer: TTCGGTACCGGAAGCTGTT
BNP	Forward Primer: GAACGTGCTGTCCCAGATGA Reverse Primer: TCCAGGAGCTTCTGCATCTT
Col1*α*	Forward Primer: GACGGGAGGGCGAGTGCTGT Reverse Primer: ACGGGTCCCCTTGGGCCTTG
Col3*α*	Forward Primer: AAAGGGTGAAATGGGTCCCAG Reverse Primer: TCACCTGAAGGACCTCGAGT
TGF‐*β*	Forward Primer: AGAAGGCAAGCCGGAGGGCA Reverse Primer: TGGGCGGGATGGCATTTTCGG
PAI‐1	Forward Primer: TGGCGTCTTCCTCCACAGCCTT Reverse Primer: GTCGGGTTGTGCCGAACCACA
cKit	Forward Primer: TCTTCCGGCACAACGGCACG Reverse Primer: GTGGGCCTGGATTTGCTCTTTGTTGT
mMCP‐1	Forward Primer: GAGCTGGAGCTGAGGAGATTA Reverse Primer: CTCAGAACCTCTGTCCGT
mMCP‐2	Forward Primer: GCACTTCTTTTGCCTTCTGG Reverse Primer: TAAGGACGGGAGTGTGGTTT
mMCP‐4	Forward Primer: TCTGGGGCTGGAGCTGAGGAGA Reverse Primer: GCAGCAGTCAACACAAATTGGCGG
mMCP‐5	Forward Primer: ATCTGCTGCTCCTTCTCCTG Reverse Primer: ACTCCGTGCCTCCAATGA
mMCP‐6	Forward Primer: GGCAGGTGAGCCTGAGATTT Reverse Primer: GGAAGAGCTGTGGGCTTTTG
CPA3	Forward Primer: CAAGGATTAAAATTGGATCAACTG Reverse Primer: GATAGCCTTTCTTTCTCCATCTTT
IL6	Forward Primer: CCTCTCTGCAGGAGACTTCCATCCA Reverse Primer: AGCCTCCGACTTGTGAGGTGGT
TNF‐*α*	Forward Primer: TACTGAACTTCGGGGTGATTGGTCC Reverse Primer: CAGCCTTGTCCCTTGAAGAGAACC
PBGD	Forward Primer: AGAAGAGCCTGTTTACCAAGGAG Reverse Primer: TTTCTCTGTAGCTGAGCCACTCT

### Statistical analysis

Data are expressed as mean ± standard error the mean (SEM). All statistics were performed using GraphPad Prism software version 5.00 for Windows (GraphPad Software Inc., San Diego, CA). Differences between parameters of control and corresponding PAB mice at each time point were compared by an unpaired two‐sided Student's *t*‐test or one‐way ANOVA followed by Dunnett's post hoc test. Differences in the time course of MRI parameters between sham and PAB mice were assessed using repeated measures ANOVA with Bonferroni post hoc test for multiple comparisons. Differences were considered statistically significant when *P* < 0.05.

## Results

In order to characterize the time course of morphological and functional changes in the RV in response to pressure overload, mice were randomly assigned to either sham or PAB group. Then a MRI analysis of the RV structure and function was performed at baseline before surgery and subsequently at 3, 7, 14, and 21 days after surgery.

**Figure 1:**
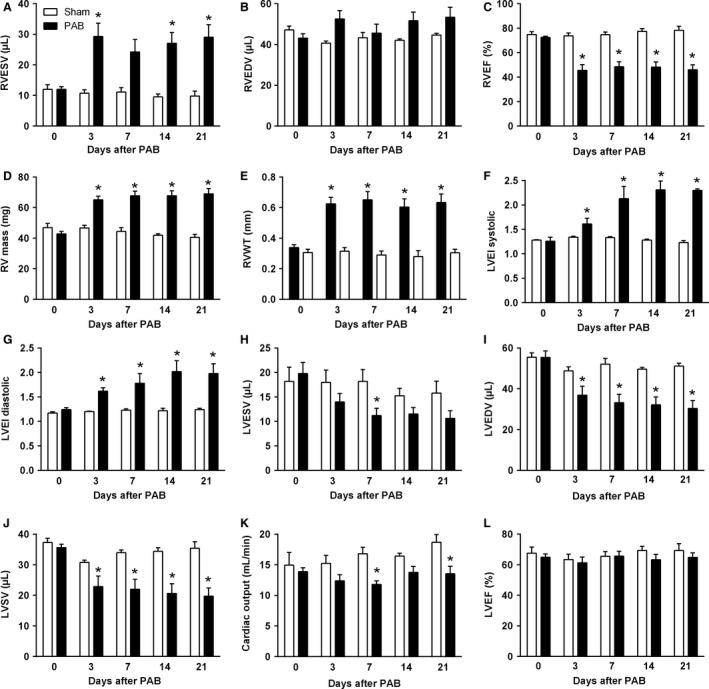
Characterization of right and left ventricular structure and function and interventricular interaction. (A) Right ventricular end‐systolic volume. (B) Right ventricular end‐diastolic volume. (C) Right ventricular ejection fraction. (D) Right ventricular mass. (E) Right ventricular wall thickness. (F) Left ventricular eccentricity index during end‐systole. (G) Left ventricular eccentricity index during end‐diastole. (H) Left ventricular end‐systolic volume. (I) Left ventricular end‐diastolic volume. (J) Left ventricular stroke volume. (K) Cardiac output. (L) Left ventricular ejection fraction. Values are means ± SEM. *P < 0.05 versus sham at corresponding time points, *n* = 10 mice per group.

Noninvasive MRI demonstrated that PAB led to severe dilatation, hypertrophy, and functional impairment of the RV (Fig. 1A–E). Pressure overload resulted in increased end‐systolic and end‐diastolic RV volumes on day 3 after PAB (Fig. 1A and B). The RV chamber dilatation was attenuated on postoperative day 7 but significantly increased afterwards. The RV dilatation was accompanied by impaired contractile function as reflected by decreased ejection fraction in PAB animals (Fig. 1C). In PAB mice, abnormal bulging of the interventricular septum toward the left ventricle due to compression exerted by dilated and hypertrophied RV, as reflected by a rapid progressive increase in the LVEI both in systole and diastole (Fig. 1F and G), led to significant reduction in left ventricular end‐systolic and end‐diastolic volumes at all time points after surgery (Fig. 1H and I). The impaired ability of the left ventricle to properly dilate and fill resulted in reduced left ventricular stroke volume and cardiac output, whereas the left ventricular ejection fraction basically remained unchanged (Fig. 1J–L).

**Figure 2:**
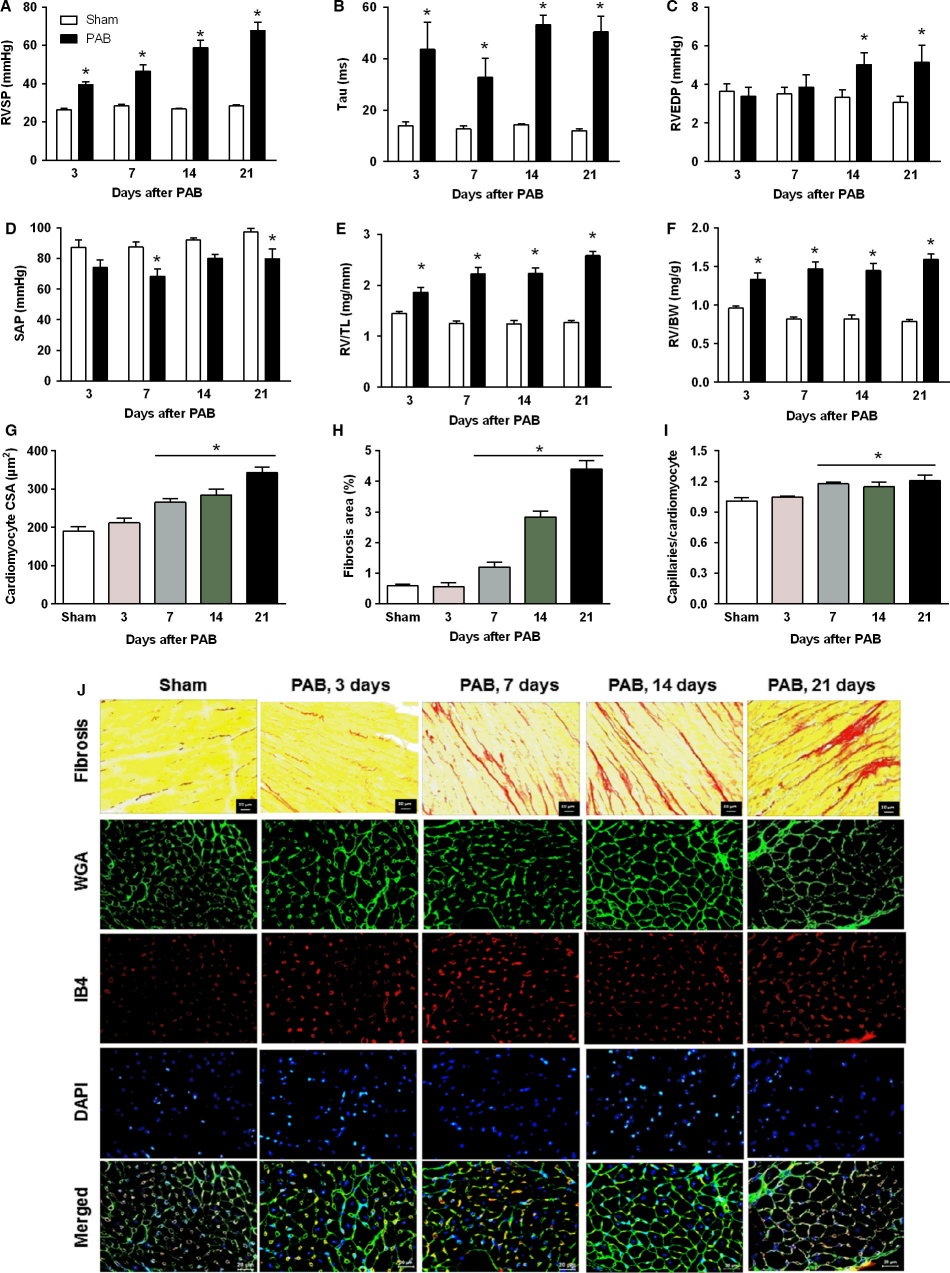
Characterization of hemodynamics and right ventricular remodeling. (A) Right ventricular systolic pressure. (B) Time constant of isovolumic relaxation of the right ventricle Tau. (C) Right ventricular end‐diastolic pressure. (D) Systemic arterial pressure. (E) Post‐mortem measured right ventricular weight values normalized to tibia length (RV/TL). (F) Post‐mortem measured right ventricular weight values normalized to body weight (RV/BW). (G) Bar graphs summarizing quantification of mean right ventricular cardiomyocyte cross‐sectional area. (H) Bar graphs summarizing quantification of interstitial fibrosis. (I) Bar graphs summarizing quantification of angiogenesis. (J) Representative images of picro‐sirius red staining of right ventricles cut in cross‐section and stained with wheat germ agglutinin‐FITC conjugate (WGA), isolectin B4‐TRITC conjugate (IB4), DAPI and merged. WGA marks cell boundaries (green), isolectin B4 (red) marks endothelial cells and DAPI marks nuclei (blue). Values are means ± SEM. *P < 0.05 vs. sham at corresponding time points, *n* = 10 mice per group for hemodynamic parameters and *n* = 5 mice per group for histological parameters.

The RVSP in PAB mice increased with time coinciding with development of RV hypertrophy (Fig. 2A). The diastolic parameter Tau was elevated in PAB mice at all time points, whereas RVEDP started increasing from day 14 (Fig. 2B and C). The systemic blood pressure decreased slightly but significantly in PAB mice (Fig. 2D). The invasively determined RV hypertrophy developed rapidly after induction of pressure overload. The RV mass significantly increased in PAB mice as early as 3 days after surgery and continued to increase thereafter until day 21 (Fig. 2E and F). On a cellular level, significant cardiomyocyte hypertrophy was evident on day 7 after PAB and gradually increased thereafter (Fig. 2G and J). Significant interstitial collagen deposition in PAB mice occurred at day 7 after surgery and progressively increased over time (Fig. 2H and J). The number of microvessels per cardiomyocyte increased significantly by day 7 and remained elevated thereafter (Fig. 2I and J).

**Figure 3:**
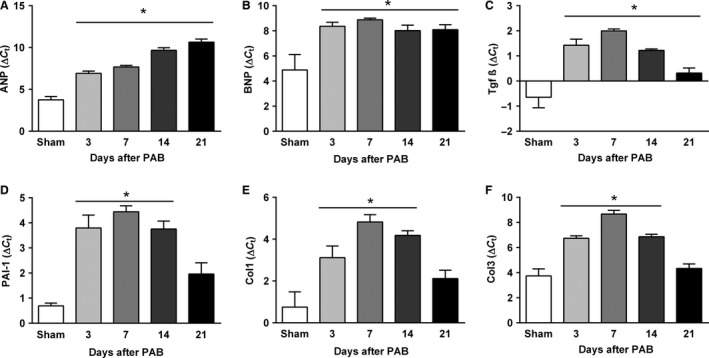
Time course of the right ventricular mRNA gene expression of hypertrophic (A and B) and profibrotic markers (C–F) in sham and PAB mice. (A) Atrial natriuretic peptide. (B) B‐type natriuretic peptide. (C) Transforming growth factor 1. (D) Plasminogen activator inhibitor‐1. (E) Collagen 1 (Col1). (F) Collagen 3 (Col 3). Values are means ± SEM. *P < 0.05 versus sham at corresponding time points, *n* = 5 mice per group. SEM, standard error the mean.

The RV remodeling process was associated with significantly increased mRNA expression levels of hypertrophic and profibrotic (ANP, BNP, collagen 1, collagen 3, Tgf‐*β,* and PAI1) markers in RV tissue from PAB mice compared with those in the sham‐operated control group. Expression of ANP and BNP mRNA increased early and was maintained at high levels up to 21 days after PAB (Fig. 3A and B). Interestingly, mRNA expression of profibrotic genes started increasing as early as 3 days after surgery, reached a maximum by day 7 and then gradually declined (Fig. 3C–F).

**Figure 4:**
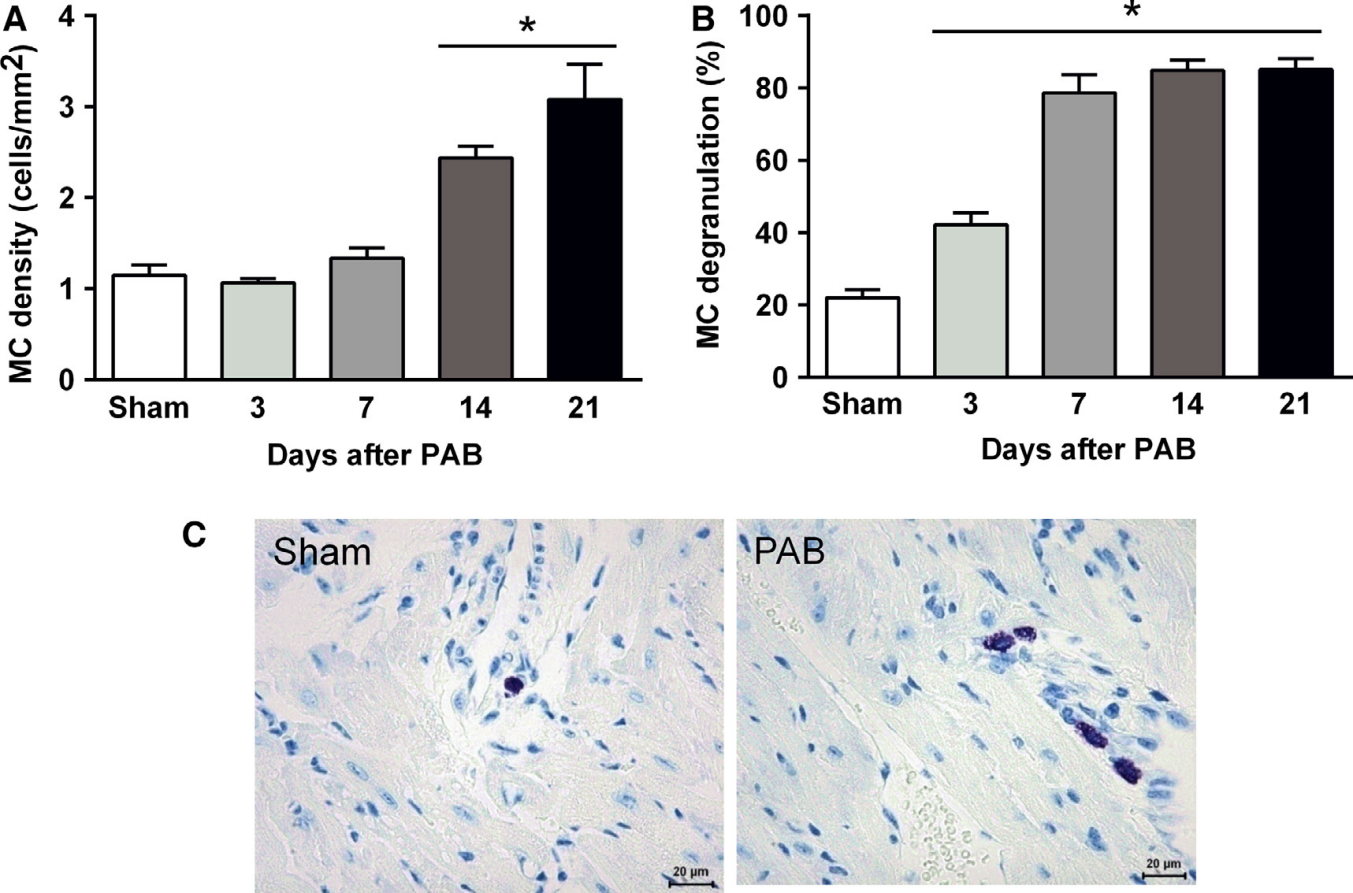
Accumulation and activation of mast cells (MC) in the process of the RV remodeling. (A) Mast cell density. (B) Mast cell activity. (C) Representative images of mast cells in RV tissue from sham and PAB mice, *n* = 5 mice per group.

**Figure 5:**
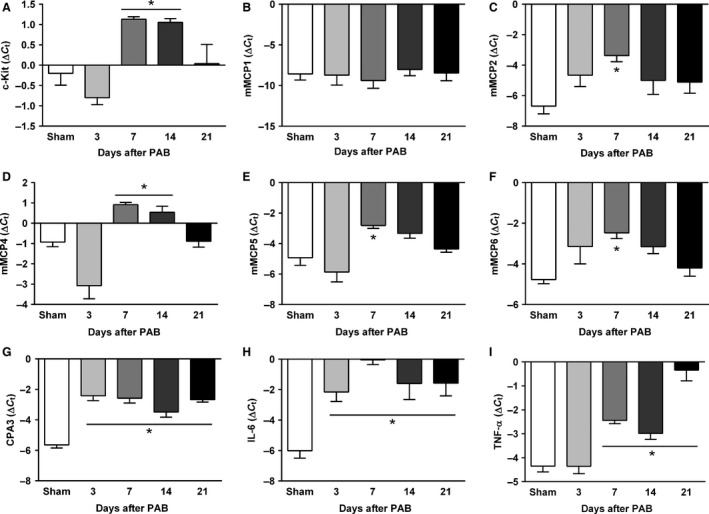
Time course of the right ventricular mRNA gene expression analysis of genes related to mast cells (A–G) and inflammation (H and I) in sham and PAB mice. (A) c‐Kit. (B) Murine mast cell protease (mMCP)‐1. (C) mMCP2. (D) mMCP4. (E) mMCP5. (F) mMCP6. (G) carboxypeptidase‐3 (CPA3). (H) IL‐6. (I) TNF‐*α*. Values are means ± SEM. *P < 0.05 versus sham at corresponding time points, *n* = 5 mice per group. PAB, pulmonary artery banding.

A time‐dependent increase in total number of mast cells and the proportion of degranulated mast cells was detected in the remodeled RV tissue from PAB mice (Fig. 4A–C). The proportion of degranulated mast cells started increasing from day 3 after PAB and reached a plateau by day 7. Remarkably, the mast cell density in PAB mice started increasing after 14 days and reached maximal values 21 days post surgery. The increased number of mast cells was accompanied by significantly increased mRNA expression of the mast cell marker c‐Kit and mast cell‐specific genes encoding murine mast cell proteases (mMCP)‐2, 4, 5, and 6 in the RV at 7 and 14 days post surgery (Fig. 5A–F). Expression of the carboxypeptidase A3 (CPA3) increased early after PAB and remained elevated for the entire period (Fig. 5G). Similarly, augmented expression of the cytokine IL‐6 was observed in RV tissue of PAB mice at all time points (Fig. 5H). Significantly elevated RV expression of TNF‐alpha in PAB mice was noted at 7 and 14 days after PAB which further increased by day 21 (Fig. 5I).

## Discussion

In this study, we characterized the longitudinal changes in RV morphology and function in response to pressure overload in C57BL/6J mice. We demonstrated that PAB led to significant dilatation, cellular and chamber hypertrophy, and functional impairment of the RV. Compression of the left ventricle exerted by dilated and hypertrophied RV caused flattening of the interventricular septum and was reflected by the increased LVEI. The latter has been shown to increase in various RV pressure and volume overload states (Ryan et al. 1985; Ascah et al. 1990; Louie et al. 1992). Due to deleterious interventricular interaction, compression of the left ventricle led to significant reduction in left ventricular end‐systolic and end‐diastolic volumes. The impaired ability of the left ventricle to properly dilate and fill resulted in reduced left ventricular stroke volume and cardiac output, whereas its contractile function remained unchanged. Progressive remodeling of the RV in PAB mice was associated with upregulation of myocardial gene markers of hypertrophy and fibrosis. Pressure overload of the RV may also lead to reduction in capillary density (Hudlicka et al. 1992), which may contribute to ventricular dysfunction. In contrast, our study demonstrated that number of capillaries per cardiomyocyte actually slightly increased in PAB mice as compared with sham mice. However, taking into account significant increase in cardiomyocyte size in PAB mice, blood supply to individual cardiomyocytes may remain reduced.

Although, pulmonary artery banding has recently become an important model of pressure overload induced RV hypertrophy and several research groups including our own have reported maladaptive alterations (Kreymborg et al. 2010; Kapur et al. 2013; Rajagopalan et al. 2013; Egemnazarov et al. 2015; West et al. 2016), serial assessment of RV morphology and function in mice subjected to pressure overload of various degrees was performed only in one study, which utilized FVB mice (Urashima et al. 2008). In that study, severe pulmonary artery stenosis was associated with significant RV cardiomyocyte and chamber hypertrophy, RV dilatation and dysfunction, reduced cardiac output, and increased mortality. Although, we have not performed a direct comparison of FVB and C57Bl/6J mice, we think that the degree of pressure overload, RV hypertrophy, and dysfunction in C57Bl/6J mice was comparable with that of FVB mice with severe pulmonary artery constriction. Our findings suggest that compared with the FVB strain, C57Bl/6J mice subjected to severe RV pressure overload are not prone to increased mortality during the first 3 weeks. The cause for increased mortality in FVB mice is not clear as RV dysfunction did not decline with time but may be related to development of malignant arrhythmias. Unfortunately, a detailed characterization of longitudinal changes in concomitant maladaptive processes, including myocardial fibrosis and capillary density alterations, is lacking for FVB mice. Nevertheless, our findings suggest that interstrain differences can affect the ability of the RV to cope with sustained pressure overload.

Inflammatory activation contributes to the pathogenesis of both left ventricular (Sun et al. 2007; Coggins and Rosenzweig 2012) and RV adverse remodeling and dysfunction (Campian et al. 2010; Rondelet et al. 2012; Dewachter et al. 2015). Previous reports have shown that cardiac mast cells are a component of the inflammatory response in various models of adverse left ventricular remodeling (Levick et al. 2008, 2009; Zhang et al. 2011). Mast cells are cells of hematopoietic lineage residing in the tissues. In normal human and rodent hearts, the number of mast cells ranges between 1.5 and 5.3 cells/mm^2^ (Rakusan et al. 1990; Patella et al. 1998). Enhanced accumulation of mast cells in left ventricular tissue from late‐stage heart failure patients (194 ± 19.6 cells/cm^2^ vs. 131 ± 22.2 cells/cm^2^ in control ventricles) has been documented (Batlle et al. 2006). Interestingly, an increase in cardiac mast cell density of similar magnitude is reported in various animal models of left ventricular hypertrophy and failure. In the rat model of left ventricular hypertrophy induced by transverse aortic constriction, the number of cardiac mast cells increased from about 2 cells/mm^2^ in sham animals to 3 cells/mm^2^ 5 weeks post surgery (Li et al. 2016). Comparable cardiac mast cell densities are reported in spontaneously hypertensive rats (Shiota et al. 2003). The temporal responses in the density of myocardial mast cells in the model of biventricular volume overload was similar for the left ventricle and RV (Brower et al. 2002). The number of mast cells in the RV at 21 days post fistula was 3.11 ± 2.01 versus 2.34 ± 1.00 cells/mm^2^ in the control. The issue of whether degranulation of this small number of cells is sufficient to cause ventricular remodeling has specifically been addressed in a study using isolated hearts, which findings clearly demonstrated that degranulation of mast cells present in normal hearts is sufficient to induce extracellular matrix degradation via activation of MMPs and produce alteration in ventricular function (Chancey et al. 2002). Importance of this small number of mast cells for cardiac remodeling is further supported by studies utilizing mast cell stabilizers, inhibitors of mast cell proteases and mast cell‐deficient mice (Hara et al. 2002; Matsumoto et al. 2003; Levick et al. 2009; Liu et al. 2012). Given the role of mast cells in the hypertensive and failing left ventricle, we were interested, if mast cells are also involved in the RV remodeling. Earlier studies in rats have shown that prolonged duration (200 days) of pressure overload on the RV induced by PAB is associated with more than threefold increase in the mast cell density (Olivetti et al. 1989). In contrast, cardiac mast cells density was not affected in 3‐month‐old rats born at high altitude despite development of significant RV hypertrophy (Rakusan et al. 1990). However, it remains unclear, if this discrepancy is due to the differences in the duration of the overload or if it is model‐dependent. In addition, temporal responses to pressure overload in the density of cardiac mast cells has not been addressed yet. We investigated the number and activity of mast cells in RV tissue at different time points after PAB. We found an early activation of mast cells evidenced by an increase in the proportion of the degranulated mast cells in the RV tissue. In contrast, mast cell density started increasing after 2 weeks and reached maximal values 3 weeks post surgery. Interestingly, increased expression of c‐Kit and soluble stem cell factor and enhanced cardiac mast cells density were observed in the prehypertensive spontaneously hypertensive rats even before any signs of cardiac hypertrophy or fibrosis (Shiota et al. 2003). An early elevation in mast cell density in both left and right ventricles was observed in a rat model of biventricular volume overload (Brower et al. 2002; Forman et al. 2006). In this model, the number of mast cells increased within 24 h and remained elevated for the first 5 days postfistula before returning to normal values by day 14.

Increase in mast cell density can result from proliferation and maturation of immature resident mast cells (Forman et al. 2006; Li et al. 2012) as well as recruitment of mast cell progenitors from the bone marrow or white adipose tissue and further proliferation and maturation in the tissue (Frangogiannis et al. 1998; Ngkelo et al. 2016). Increased chymase activity secondary to mast cell activation may increase tissue levels of stem cell factor by cleaving membrane‐bound stem cell factor to release a soluble form (Longley et al. 1997). Higher levels of the stem cell factor then rapidly elevate mast cell numbers by stimulating maturation of immature resident mast cells in the heart (Li et al. 2012). However, the mast cell density returns to normal values at 5–7 days post fistula (Brower et al. 2002; Forman et al. 2006). Thus, the increase in cardiac mast cell density in response to volume overload is due to maturation and differentiation, but not proliferation of a resident population of immature cardiac mast cells. Rather late increase in mast cell density in our experiments may be due to different underlying mechanisms. Indeed, recruitment of mast cell precursors and further maturation in the tissue requires a considerable amount of time (Jamur et al. 2010). In addition, interspecies differences (Halapas et al. 2008) and type of overload stress (Bartelds et al. 2011; Borgdorff et al. 2013) may account for this discrepancy. Nevertheless, this issue deserves further investigation.

Depending on the magnitude of the stimulus, activation mode and selective activation of specific signaling pathways, mast cells release unique arrays of preformed and newly synthesized biologically active substances (Rao and Brown 2008; Gilfillan et al. 2011). Potential mechanisms of the involvement of mast cells in the cardiac remodeling include stimulation of collagen synthesis leading to myocardial fibrosis or activation of matrix metalloproteinases resulting in collagen degradation and ventricular dilatation. Mast cells serve as a source of a number of cytokines, which are mitogenic and chemotactic for fibroblasts and stimulate production of extracellular matrix by fibroblasts. It has been demonstrated that cardiac mast cells participate in the induction of cardiac hypertrophy and cardiac fibrosis by synthesizing and secreting prohypertrophic cytokines and profibrotic growth factors TGF‐*β* and bFGF (Shiota et al. 2003). Moreover, cardiac mast cells can promote tissue fibrosis by stimulating cell proliferation and collagen expression in cardiac fibroblasts through PDGF‐A (Liao et al. 2010). Mast cells also release both preformed and newly synthesized cytokines TNF‐*α* and IL‐6 (Gordon and Galli 1990, 1991; Gagari et al. 1997) which have previously been shown to mediate cardiac fibrosis and hypertrophy in various animal models (Sun et al. 2007; Melendez et al. 2010; Gonzalez et al. 2015; Sriramula and Francis 2015). Similar to the findings from the left ventricular hypertrophy studies, we found significantly elevated mRNA levels of TNF‐*α* and IL‐6 in RV tissues from PAB mice compared with those from sham mice suggesting their potential contribution to RV remodeling process. Besides cytokines, mast cell granules contain very high levels of a number of proteases and mast cell degranulation leads to release of active mast cell proteases (Stevens and Adachi 2007). Mouse mast cell consists of various proteases including chymases (mMCP‐1, ‐2, ‐4, ‐5, ‐9), tryptases (mMCP‐6, ‐7, membrane‐bound tryptase and a number of other trypsin‐like serine proteases), and the exopeptidase CPA3 (Pejler et al. 2010). Mast cell chymases and tryptases are potent activators of fibroblast proliferation and inducers of matrix protein synthesis (Cairns and Walls 1997; Akers et al. 2000; Zhao et al. 2008; McLarty et al. 2011). Mast cells proteases can promote extracellular matrix accumulation through formation of angiotensin II (Urata et al. 1990), activation of TGF‐*β* (Lindstedt et al. 2001), or by acting as mitogens for fibroblasts (Ruoss et al. 1991). Previous studies have demonstrated that expression and activity of mast cell proteases tryptase and chymase are elevated in the hypertensive left ventricles (Shiota et al. 1997; Li et al. 2002; Levick et al. 2009; Li et al. 2016). We performed analysis of gene expression in RV tissues from PAB mice and found a significant upregulation of several mouse mast cell proteases including mMcp‐2, 4, 5, 6, and CPA3. Inhibition of mast cell chymase and tryptase has been shown to prevent cardiac fibrosis and improve left ventricular dysfunction in various left heart disease models (Matsumoto et al. 2003; Kanemitsu et al. 2006, 2008; Li et al. 2016). Interestingly, inhibition of mast cell chymase increases the cardiac efficacy of angiotensin I‐converting enzyme inhibitor therapy and improves survival after myocardial infarction in hamsters (Jin et al. 2003; Wei et al. 2010). These data demonstrate that selective inhibition of mast cell proteases might represent an important strategy for management of cardiac dysfunction.

In summary, our work provided a detailed description of the longitudinal changes in RV morphology and function in response to pressure overload in C57BL/6J mice. We also demonstrated accumulation and activation of mast cells in the RV tissue of PAB mice. Our data suggest possible involvement of mast cells in the RV remodeling process in response to pressure overload. Mast cells may thus represent an interesting target for the development of new therapeutic approaches directed specifically at the RV.

## Conflicts of Interest

No conflicts of interest, financial or otherwise, are declared by the author(s).
